# The Art of War: harnessing the epigenome against cancer

**DOI:** 10.12688/f1000research.12833.1

**Published:** 2018-02-02

**Authors:** Jonathan Nye, Daniël P. Melters, Yamini Dalal

**Affiliations:** 1 Center for Cancer Research, National Cancer Institute, National Institutes of Health, Bethesda, MD, USA

**Keywords:** Epigenome, Histone chaperones, chromatin, cancer, tumor growth

## Abstract

Histone chaperones are indispensable regulators of chromatin structure and function. Recent work has shown that they are frequently mis-regulated in cancer, which can have profound consequences on tumor growth and survival. Here, we focus on chaperones for the essential H3 histone variants H3.3 and CENP-A, specifically HIRA, DAXX/ATRX, DEK, and HJURP. This review summarizes recent studies elucidating their roles in regulating chromatin and discusses how cancer-specific chromatin interactions can be exploited to target cancer cells.

## Introduction

Histones are a highly conserved family of proteins that facilitate the compaction of DNA by wrapping it around an octamer containing two copies each of the canonical histones H2A, H2B, H3, and H4. These canonical forms comprise the large majority of all histones bound to DNA and are responsible for the regulation of a variety of cellular processes including replication, transcription, and DNA repair. In addition, several histone variants have evolved to allow for additional levels of regulation. These variants can differ from their canonical counterparts in sequence, structure, and the timing of their incorporation. Canonical histone assembly is typically coupled to DNA replication at S-phase, whereas the assembly of histone variants is replication independent and spans all phases of the cell cycle ^1^.

**Figure 1.**
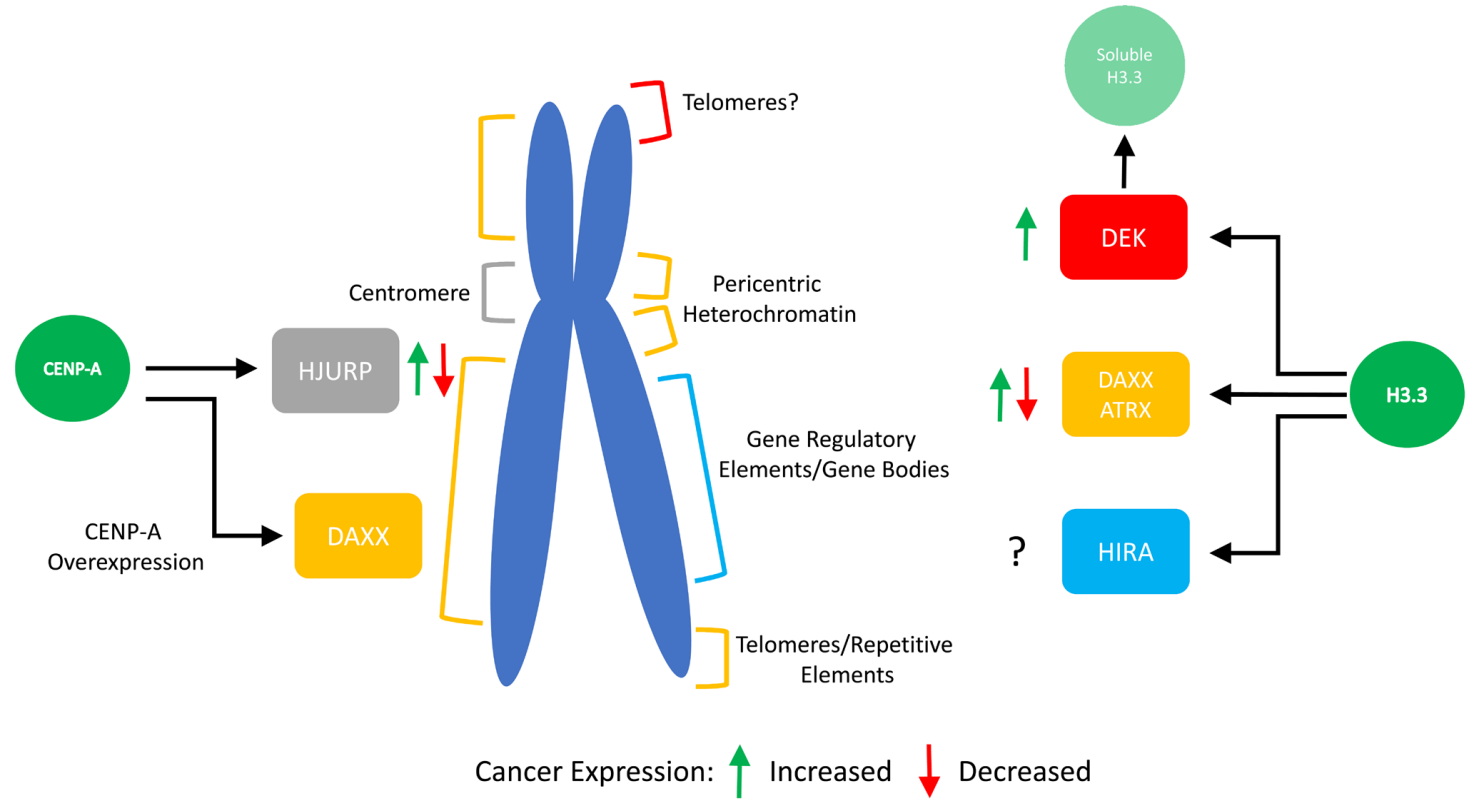
Histone chaperones allow for assembly at specific genomic regions. The histone variant H3.3 relies on three specific chaperones for deposition at specific locations in the genome. The chaperones DEK, DAXX/ATRX, and HIRA have been shown to prefer distinct sites for H3.3 assembly. Furthermore, they have been found to be mis-regulated in many cancer types, as shown. The centromeric histone variant CENP-A normally associates with a single chaperone called HJURP. However, changes in the amount of CENP-A compared to its chaperone can allow for deposition throughout chromosome arms by the H3.3 chaperone DAXX.

Human histone variants have been identified for all canonical histones except for H4. The canonical histone H3 has six variants including H3.3, CENP-A, H3.1T, H3.5, H3.X, and H3.Y ^2^. This diversity allows for variants that specialize in a wide variety of different functions, including the regulation of transcription, chromosome segregation, and telomere function. Interestingly, the two most abundantly expressed and essential H3 variants differ not only in function but also in how much they have diverged from the canonical form. For example, the H3 variant H3.3 differs by five amino acids and shares 96.3% amino acid sequence similarity with its canonical counterpart H3.1; in contrast, CENP-A exhibits only 45.1% similarity with H3.1 ^2^. Furthermore, while their assembly is replication-independent, they localize to distinct regions of the genome: CENP-A is normally found predominantly at the centromere, whereas H3.3 localizes to heterochromatin, telomeres, enhancers, and genic areas of high nucleosome turnover ( Figure 1).

The precise localization and assembly of these histones into chromatin is thought to be achieved through their interaction with histone variant specific chaperones by mechanisms not yet entirely elucidated. In addition, some chaperones are found to be closely associated with ATP-dependent chromatin remodelers. For example, proper H3.3 assembly and localization relies on the histone chaperone DAXX in complex with the SWI/SNF-like chromatin remodeler ATRX. Interestingly, H3.3 can associate with multiple chaperones, including HIRA and DEK, in addition to DAXX/ATRX. In contrast, human CENP-A normally associates with a single centromeric chaperone called HJURP. Intriguingly, while it has long been assumed that histone chaperones are mere carriers of histones, recent advances, including patient tumor sequencing data, have shown that these critical chaperones may play an unanticipated role in disease progression. Here, we review recent literature on this subject and ask how changes in variant chaperones can influence the histone variant chromatin landscape in the epigenome and thereby impact human health.

## HIRA and senescence

The histone cell cycle regulator (HIRA) protein has been found to be a chaperone facilitating the assembly of the histone variant H3.3 into chromatin in a replication-independent manner ^3^. It is at the center of a complex of proteins, conserved from yeast to humans, referred to as the HUCA complex that consists primarily of HIRA, UBN1, CABIN1, and transiently includes Asf1 ^4^. The UBN1 subunit imparts specificity to the complex by preferentially binding to H3.3/H4 over H3.1/H4, thereby enabling this dimer’s assembly into chromatin ^5^. Unlike other H3 variants like CENP-A, H3.3 normally associates with multiple chaperones and each complex appears to be responsible for its localization to specific places in the genome. For example, HIRA is necessary for H3.3 deposition at gene regulatory elements, gene bodies, and sites of DNA damage ^6, 7^. Targeting of the HIRA complex is thought to be achieved through its interaction with a number of different proteins including transcription factors, chromatin remodelers, and the single-stranded DNA (ssDNA)-binding protein RPA ^6, 8^. However, the precise mechanism by which this chaperone hones in on the correct regions of the genome has not been clearly elucidated. One possibility is that HIRA recruitment to gene regulatory regions requires the presence of R-loops. These RNA–DNA hybrid structures attract RPA owing to the presence of exposed ssDNA, which then recruits the HIRA complex and leads to H3.3 assembly at these sites. From these studies, it is clear that the HIRA complex is an important regulator of H3.3 deposition, but HIRA’s role in human health is still unclear.

This is particularly intriguing because human HIRA was originally identified through the study of DiGeorge syndrome patients, who commonly have heart and brain abnormalities, arising from a deletion of the q11 cytogenetic band of chromosome 22, which contains the HIRA gene ^9, 10^. Despite intense study, it is still unclear as to whether HIRA is responsible for these defects or if it is a result of the deletion of multiple genes. More recently, HIRA has been shown to have a clear role in establishing and maintaining senescence. Indeed, early studies discovered that HIRA and Asf1 are necessary for the formation of senescence-associated heterochromatic foci, which, in turn, are thought to be essential to shut down genes involved in cell cycle progression ^11^. Furthermore, the overexpression of HIRA and Asf1 is sufficient to induce senescence. In addition, post-translational modifications of HIRA have been identified and were shown to be necessary for its function. Consistent with HIRA’s proposed role, the expression of a non-modifiable mutant led to defects in senescence ^12^. Multiple mechanisms have been proposed to explain the role of HIRA in the establishment of senescence. One provocative possibility involves a cleaved H3.3 protein lacking the first 21 amino acids. HIRA-mediated assembly of this cleaved protein has been shown to be sufficient to induce senescence and results in the repression of cell cycle regulators ^13^. Other work has reported that HIRA is necessary for replication-independent deposition of H3.3 in senescent cells and for maintaining the H4K16ac histone mark at gene promoters ^14, 15^. Furthermore, the same study found that HIRA was required to suppress oncogene-induced neoplasia in a mouse model. This work highlights the importance of this H3.3 chaperone in fine tuning the chromatin environment to allow cells to permanently exit the cell cycle and preventing uncontrolled cell growth. As discussed in the next section, many questions remain unanswered: for instance, how is HIRA function affected by changes in levels of another H3.3 chaperone, DAXX, commonly observed in tumors? For example, can HIRA bind to other H3 variants as has been observed with DAXX? Furthermore, in Arabidopsis, H3.3 and DNA methylation are inversely related and H3.3 knockdown alters the DNA methylation profile ^16, 17^. Moreover, it has been proposed that H3.3 prevents the recruitment of the linker histone H1 ^18^. These findings beg the following questions: can global changes in DNA methylation, coupled to widespread mis-regulation of linker histone H1 isoforms, both commonly associated with cancer, alter H3.3 deposition? Or, conversely, does gain or loss of H3.3 at a specific locus alter its DNA methylation profile? Both of these fundamental questions need to be addressed in future studies.

## H3.3 deposition by DAXX/ATRX in cancer

**Figure 2.**
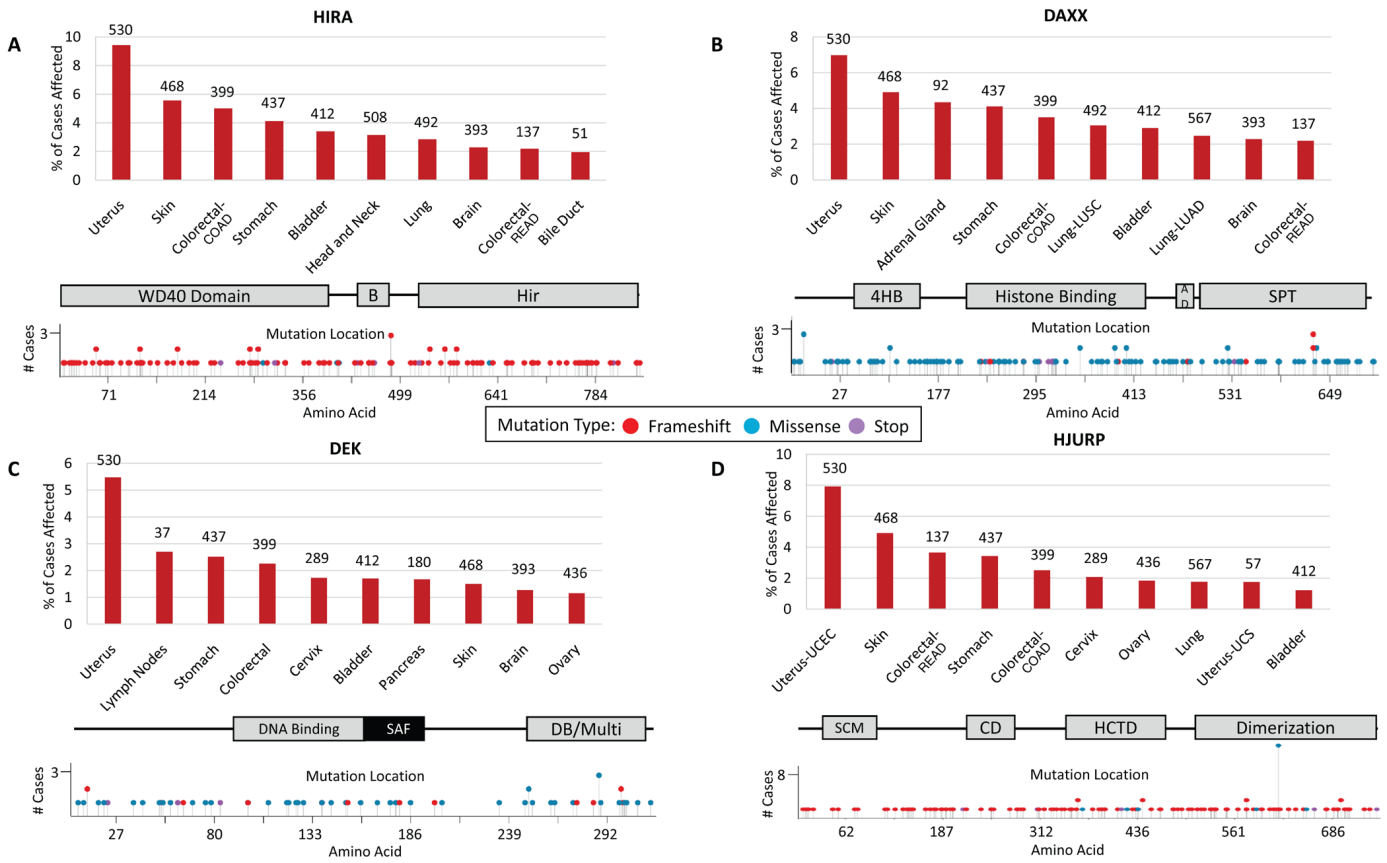
Mutations in H3 variant chaperones occur in many cancer types. **A**. Percentage of cases affected by HIRA mutations in multiple cancer types obtained from The Cancer Genome Atlas (TCGA) analysis. Numbers listed above each bar represent total number of cases analyzed. TCGA cancer types listed in order from left to right include uterine corpus endometrial carcinoma (UCEC), skin cutaneous melanoma (SKCM), colon adenocarcinoma (COAD), stomach adenocarcinoma (STAD), urothelial bladder carcinoma (BLCA), head–neck squamous cell carcinoma (HNSC), lung squamous cell carcinoma (LUSC), glioblastoma multiforme (GBM), rectum adenocarcinoma (READ), and cholangiocarcinoma (CHOL). Locations of mutations within the HIRA protein. HIRA protein domains include a WD-40 repeat-containing domain, the B-domain (B) necessary for binding to Asf1, and the conserved Hir domain. **B**. DAXX mutations as above. TCGA cancer types listed in order from left to right include UCEC, SKCM, adrenocortical carcinoma (ACC), STAD, COAD, LUSC, BLCA, lung adenocarcinoma (LUAD), GBM, and READ. DAXX protein domains include the four-helix bundle (4HB) necessary for ATRX binding, a histone-binding domain, an acidic domain, and the Ser/Pro/Thr-rich region. **C**. DEK oncogene mutations listed as above. TCGA cancer types listed in order from left to right include UCEC, diffuse large B-cell lymphoma (DLBC), STAD, COAD, cervical squamous cell carcinoma and endocervical adenocarcinoma (CESC), BLCA, pancreatic adenocarcinoma (PAAD), SKCM, GBM, and ovarian cancer (OV). DEK protein domains shown include a DNA-binding domain, the scaffold attachment factor-box (SAF), and a DNA-binding and multimerization domain. **D**. HJURP mutations listed as above. TCGA cancer types listed in order from left to right include UCEC, SKCM, READ, STAD, COAD, CESC, OV, LUAD, uterine carcinosarcoma (UCS), and BLCA. HJURP protein domains include the SCM3 domain, the conserved domain (CD), the HJURP C-terminal domain (HCTD) responsible for centromere targeting, and the dimerization domain.

The histone chaperone DAXX, or death domain-associated protein, was originally named for its association with the Fas receptor, wherein it was thought to induce apoptosis by activating the JNK pathway ^19^. However, further work identified it as a bona fide H3.3 chaperone that forms a complex with the SWI/SNF-like chromatin remodeler ATRX ^20– 22^. Like other H3.3 chaperones, ATRX/DAXX targets H3.3 to very specific regions of the genome in a replication-independent manner, specifically, to telomeres, pericentric heterochromatin, and other repetitive elements ^23– 25^. Both ATRX and DAXX seem to be equally important in this process, with DAXX providing the H3.3 binding specificity and chaperone activity while ATRX targets the complex in part through binding to modified histones like H3K9me3 and also stretches of G-rich repeats with a unique secondary DNA structure called a G-quadruplex ^26– 30^. Moreover, proper functioning of this complex is critical, since mutations in both of these proteins have been strongly linked to cancer and other diseases ( Figure 2B, Table 1).

**Table 1.** Expression changes of histone variant chaperones in cancer. ALT, alternative lengthening of telomeres; GBM, gliobastoma multiforme.

Histone Variant	Chaperone/Chromatin Remodeler	Cancer Expression Level	Functional Consequences	Refs
H3.3	HIRA	?	Decreased levels prevent senescence and increase oncogene-induced neoplasia in mouse model	14
	DAXX	Increased	Promotes tumor growth in mouse prostate cancer model Increased expression of oncogenes in GBM cells lacking PTEN Promotes mis-localization of CENP-A, leading to chromosomal instability Promotes proliferation and resistance to anticancer treatments	39 38 40– 42 43
		Decreased	Potentiates Slug-driven lung cancer metastasis	37
	ATRX	Decreased	Activation of ALT pathway	36
	DEK	Increased	Upregulates anti-apoptotic factors Fusion protein dominant negative for DEK function Increased colony formation and tumorigenesis	44 45– 50 51
CENP-A	HJURP	Increased	?	52– 60
		Decreased	Chromosomal instability	61, 62

ATRX was first discovered by identifying mutations in patients with an inherited disorder, ATRX syndrome, which resulted in a wide array of developmental defects ^31^. Since then, mutations in both ATRX and DAXX have been found in a variety of different tumor types and seem to be especially prevalent in tumors associated with the central nervous system ^32– 35^. For example, in pediatric glioblastoma multiforme (GBM), 31% of patients have mutations in either ATRX or DAXX ^36^. It is unclear how these mutations are driving cancer in young patients, but it is likely that ATRX and DAXX deficiencies have adverse effects on chromatin structure that may contribute to the development of cancer. As has been noted in patient tumors, ATRX/DAXX deficiency is commonly associated with alternative lengthening of telomeres (ALT) pathway activation, in which telomere length is maintained in a telomerase-independent manner, allowing cell growth to continue on uncontrolled ^37^. Moreover, DAXX has also been shown to suppress lung cancer metastasis driven by the transcription factor Slug, directly binding to it, sequestering it, and preventing its association with DNA. Consequently, low DAXX expression levels correlated with lower overall survival in lung cancer patients with Slug expression ^38^.

While these studies conclude that DAXX activity may serve a cancer-protective function, recent work has shown, conversely, that the presence of DAXX in some cases may *enhance* tumor growth as well as generate resistance to treatment. For example, in GBM cells lacking the tumor suppressor PTEN, there is a DAXX-dependent increase in the expression of oncogenes, and inhibition of DAXX in this context can suppress tumor growth ^39^. DAXX has also been found to promote tumor growth in a mouse xenograft model of prostate cancer ^40^. Furthermore, ONCOMINE meta-analysis revealed that overexpression of DAXX is common in prostate cancer patients and correlates with lower survival rates, suggesting that, in certain cases, DAXX may present itself as a viable therapeutic target.

One possibility is that chaperones might, in the cancer background, bind inappropriately to the wrong histone variant. Indeed, recent studies have shown that DAXX can bind to the centromeric histone variant CENP-A, which is naturally overexpressed in colorectal cancer cells. Indeed, both DAXX and ATRX are present at severalfold excess in these cells ^41^. These data suggest that serendipitous overexpression of chaperones alongside non-target histone variants might promote their association and drives mis-localization. Interestingly, this mis-localization has been shown to lead to genomic instability and also greater resistance to anticancer treatments ^42, 43^. These findings may provide a mechanistic explanation for earlier data showing that DAXX appears to promote proliferation and chemoresistance in ovarian cancer cells ^63^.

From these studies, it is clear that the roles of DAXX and ATRX in cancer are complex and dependent on many factors, including the accompanying mutations, the tumor type, and alterations in histone variant expression. In the future, it will be important to understand exactly how changes in DAXX expression can drive cancer progression. It is likely that cells rely on a delicate balance between different chaperones. It will be interesting to test whether the phenotypes observed when DAXX is overexpressed are due to a titration of H3.3 *away* from HIRA, resulting in mis-regulation of gene regulatory elements, or defects in senescence, both thought to be controlled by that chaperone. If so, this would support a “chaperone competition” model, in which changes in chaperone expression lead to widespread changes in localization of their target histone variants and binding of chaperones to non-cognate partners, thereby potentially driving tumorigenesis.

## The proto-oncogene DEK

In addition to HIRA and DAXX, another H3.3 chaperone exists, namely the proto-oncogene DEK ^64^. DEK has been implicated in a wide variety of cellular processes including transcription, replication, and DNA repair ^65^. Interestingly, DEK also has the unique ability to bind preferentially to four-way junction DNA and induce positive supercoiling ^66, 67^. In its role as an H3.3 chaperone, it has been shown to play a critical role in regulating the deposition of H3.3 by HIRA and ATRX/DAXX ^68^. Depletion of DEK in embryonic stem cells leads to the promiscuous incorporation of H3.3 throughout chromosome arms and pericentric heterochromatin by HIRA and DAXX. However, H3.3 is lost from telomeric chromatin and results in telomere dysfunction. Thus, DEK seems to behave as a gatekeeper, maintaining a balance between soluble and chromatin-bound H3.3 by modulating access to H3.3 by different chaperones.

As with the other chaperones, DEK overexpression can be found in many cancer types and correlates with increased proliferation and tumorigenesis ( Table 1) ^42, 44, 69– 74^. DEK may promote tumorigenesis in multiple ways. First, it has been shown to prevent apoptosis by upregulating anti-apoptotic factors ^45^. Consequently, reducing DEK levels led to increased apoptosis and susceptibility to genotoxic agents in melanomas. Second, in acute myeloid leukemia, DEK has been reported to be the target of translocations that generate a fusion protein with NUP214 ^46– 50^. Precisely how this contributes to tumorigenesis is unclear; however, this fusion protein has been shown to interact with wild-type DEK and also inhibits chaperone activity ^64^. Third, DEK overexpression has been shown to inhibit senescence ^63^. Indeed, DEK levels are reduced upon replicative senescence, and overexpression of the protein leads to prolonged lifespan. Consequently, DEK-knockout mice develop fewer tumors in a chemical carcinogenesis model, while overexpression leads to increased colony formation and tumorigenesis ^75^.

Since DEK is involved in so many different pathways, it will not be trivial to pin down exactly how, or whether, its overexpression promotes tumorigenesis via histone variant assembly pathways. One possibility involves its regulation of H3.3 assembly by HIRA and DAXX. Normally, DEK seems to function to counteract the assembly of H3.3 by these two complexes by maintaining the soluble pre-nucleosomal H3.3 pool ^68^. Thus, overexpression may prevent the proper assembly of this important histone variant by sequestering away H3.3 from HIRA or from DAXX. Indeed, it has been shown that depletion of HIRA leads to defects in senescence ^11– 14^, which has also been shown in cases of DEK overexpression ^51^.

## The centromeric chaperone HJURP

The centromere-specific histone H3 variant CENP-A/CENH3 is the epigenetic mark that specifies the site for kinetochore assembly during mitosis ^76^. This allows for proper microtubule attachment to the chromosome and facilitates proper segregation of sister chromatids during anaphase. Like the H3.3 histone variant, CENP-A deposition occurs in a replication-independent manner. In human cells, the histone chaperone HJURP is responsible for its deposition during late mitosis/early G1 ^61, 77– 78^. HJURP localization and licensing is tightly linked to cell cycle progression and requires phosphorylation by CDK/cyclin A ^79, 80^. Both in the soluble preassembly complex and on chromatin, HJURP forms a homodimer ^81^. Chromatin-bound HJURP is lost from centromeric chromatin by late G1/early S phase ^52^, while a trace amount of it appears to return after replication, which may ensure that CENP-A can be deposited once and only once per cell cycle.

Like most other histone chaperones, the overexpression of HJURP has been observed in various cancers ^53– 60^. In particular, breast, liver, and prostate cancer mis-regulate 14 centromere and kinetochore genes, including HJURP ^82^; this combinatorial mis-regulation has been proposed as a prognostic and predictive marker. These data also provide tantalizing functional links between cancer progression and mis-regulation of centromere chromatin because half of the 14 mis-regulated genes were found to be involved in the directed assembly of CENP-A nucleosomes. Interestingly, recent work has shown that the tumor suppressor p53 binds to elements in the promoters of CENP-A and HJURP and serves to repress the expression of these genes. Thus, loss of p53, a common phenomenon in cancer, can result in the overexpression of HJURP and CENP-A ^62^. Indeed, in colon cancer cells with a mutated *p53* gene, a DNase I hotspot maps to the CENP-A promoter, suggesting enhanced transcription of this gene, and correlates with increased RNA and protein levels of CENP-A ^41^. This might explain why various types of tumors overexpress HJURP and CENP-A. In addition, a SNP located in the HJURP gene was found to be associated with increased risk for hepatocellular carcinoma among a Chinese population that were infected with hepatitis B virus ^83^. This correlated with a *decrease* in expression of HJURP at the mRNA and protein level.

These observations raise several questions. First, can changes in HJURP expression observed in cancer result in ectopic deposition of CENP-A? Indeed, in various cancers, ectopic CENP-A has been observed ^41, 84^, and the overexpression of CENP-A has been shown to be sufficient for ectopic localization ^42, 43^. Furthermore, this ectopic localization of CENP-A can result in chromosome instability. As noted above, CENP-A is deposited ectopically not by HJURP but by the H3.3 chaperone DAXX. This would suggest that when HJURP is limiting, CENP-A can bind promiscuously to other chaperones, allowing for ectopic localization ^85^. In addition, ectopic CENP-A nucleosomes can form highly stable heterotypic CENP-A/H3.3 nucleosomes ^41, 42, 86^. The origin, and consequences, of these heterotypic hybrid nucleosomes *in vivo* is a focus of intense studies. One possibility is that CENP-A is able to associate directly with DAXX but requires HJURP as an intermediate chaperone. Another possibility is that upon HJURP overexpression, DAXX and HJURP can form a heterodimer analogous to the HJURP homodimer, leading to the formation of these heterotypic nucleosomes ^81^. Finally, in high-turnover regions, H3.3 nucleosomes are thought to ‘split’ in half during transit of RNA polymerases ^87– 89^, providing a tantalizing means for invasion of an existing H3.3 nucleosome by a dimer of CENP-A/H4.

Second, can mutations in CENP-A’s protein sequence, or in the gain or loss of specific post-translational modifications, alter its affinity for HJURP in cancer? Recently, a modification of CENP-A through acetylation and ubiquitination of lysine 124 as well as phosphorylation of serine 68 was discovered ^52, 90– 93^. Interestingly, ubiquitination of K124 and phosphorylation of S68 seem to play antagonistic roles, the first being necessary for HJURP binding and the latter inhibiting it, resulting in enhanced ectopic localization ^93, 94^. In addition, recent work has shown that when the tumor suppressor Fbw7 is lost, CENP-A S18 becomes hyper-phosphorylated, leading to chromosomal instability and tumor progression as a result of reduced CENP-A at centromeres ^95^. It is clear from this work that the proper localization and function of CENP-A relies on modifications that either enhance or reduce its affinity for the chaperone HJURP. However, it is also likely that cancer cells exploit these pathways to promote tumor growth. In the future, it will be interesting to investigate whether this occurs for other H3 variants as well. For example, pre-assembly H3.3/H4 heterodimers have also been shown to be modified, but it is unclear whether these modifications are altered in tumors ^96, 97^.

## Conclusion

A major problem when trying to identify specific mechanisms that promote tumorigenesis is that even seemingly subtle changes, such as point mutations in a chaperone ( Figure 2), can dramatically alter the epigenetic landscape of a tumor ^36^. Therefore, in the battle against cancer, the enemy has an apparent advantage. Indeed, in this regard, cancer cells appear to have mastered the maxim, “be extremely subtle, even to the point of formlessness... thereby you can be the director of the opponent's fate” ( Sun Tzu, *The Art of War*).

The crucial question is whether the chaperone–histone mis-interactions listed above, driven by mutation, mis-expression, or mis-regulation, can serve as therapeutic targets in the treatment of disease ^98^. What makes such interactions an attractive target is precisely that they do not exist in normal cells. Thus, there are likely a small set of critical interactions that might be meaningful to exploit. Consequently, it will be informative to test whether identifying and blocking cancer-specific interactions between histone variants and chaperones, such as DAXX binding to CENP-A, or between chaperones and chromatin regulatory complexes can serve as a potent method to singularly attack cancer-specific networks while sparing normal cells. In this quest, using advanced techniques such as molecular docking, computational modeling, sophisticated machine-learning algorithms to query mutated protein interactomes, and focused small molecule design to identify and disrupt local affinities in protein–protein or DNA–protein interactions presents exciting and promising avenues of research. Thus, in our battle against disease, we note another maxim that promises hope: "in the midst of chaos, there is also opportunity” (Sun Tzu, *The Art of War*).
